# CAWE-ACNN Algorithm for Coprime Sensor Array Adaptive Beamforming

**DOI:** 10.3390/s24175454

**Published:** 2024-08-23

**Authors:** Fulai Liu, Wu Zhou, Dongbao Qin, Zhixin Liu, Huifang Wang, Ruiyan Du

**Affiliations:** 1 Laboratory of GNSS Anti-Jamming Technology, Northeastern University at Qinhuangdao, Qinhuangdao 066004, China; ruiyandu@126.com; 2 Hebei Key Laboratory of Marine Perception Network and Data Processing, Northeastern University at Qinhuangdao, Qinhuangdao 066004, China; 3 School of Computer Science and Engineering, Northeastern University, Shenyang 110819, China; 4 School of Electrical Engineering, Yanshan University, Qinhuangdao 066004, China; lzxauto@ysu.edu.cn

**Keywords:** robust adaptive beamforming, attention convolutional neural network, weight vector estimation, coprime sensor array

## Abstract

This paper presents a robust adaptive beamforming algorithm based on an attention convolutional neural network (ACNN) for coprime sensor arrays, named the CAWE-ACNN algorithm. In the proposed algorithm, via a spatial and channel attention unit, an ACNN model is constructed to enhance the features contributing to beamforming weight vector estimation and to improve the signal-to-interference-plus-noise ratio (SINR) performance, respectively. Then, an interference-plus-noise covariance matrix reconstruction algorithm is used to obtain an appropriate label for the proposed ACNN model. By the calculated label and the sample signals received from the coprime sensor arrays, the ACNN is well-trained and capable of accurately and efficiently outputting the beamforming weight vector. The simulation results verify that the proposed algorithm achieves excellent SINR performance and high computation efficiency.

## 1. Introduction

Adaptive beamforming is a critical task in sensor array signal processing, which enables a high directional gain towards desired signals while suppressing interference signals, so it has been widely applied in wireless communication [1], radar [2] and sonar [3]. The minimum variance distortionless response (MVDR) beamforming algorithm has an excellent signal-to-interference-plus-noise ratio (SINR) performance when the interference-plus-noise covariance matrix (INCM) is available and the steering vector (SV) of the desired signal is known exactly [4]. Regrettably, in practical environments, some unexpected errors caused by sensor position and direction of arrival (DOA) estimation cannot be avoided, which can result in a mismatched SV, thereby reducing the SINR [5]. To improve the robustness of the MVDR algorithm, a sequential quadratic programming (SQP)-based method is presented to correct the SV of the desired signal through a quadratic convex optimization problem [6]. It achieves a better output SINR performance, for it successfully solves the SV mismatch problem of the uniform linear array (ULA).

For the ULA, it has a low installation cost in practical applications, given its simple structure. However, the distance between arrays is usually maintained at less than 1/2 the wavelength to accurately estimate the signal, which will lead to signals from different arrays being influenced by each other, and the estimated performance will decrease [7]. At the same time, due to the limitations of hardware costs and power consumption, it is unreasonable to increase the array aperture for resisting interference by increasing the number of sensors [8]. In this case, the coprime array is proposed, which has a larger array aperture and higher degrees of freedom (DOF) than the ULA in the case of not increasing the number of sensors, which helps to improve the interference suppression capability and weaken the mutual coupling effects between different arrays [9]. An INCM reconstruction-based adaptive beamforming algorithm is presented for coprime sensor arrays [10]. Specially, the DOAs of each signal can be estimated by a pair of uniform linear subarrays, which are obtained via decomposing the coprime sensor array. Subsequently, the estimated DOAs are utilized to reconstruct the INCM and estimate the desired signal SV. The simulation results verify the robustness of the proposed algorithm in different environments, but it has limited real-time performance due to the process of spectral searching. In addition, a coprime virtual uniform linear array (CV-ULA) is given by deducing the virtual sensors. After that, the Toeplitz matrix of the CV-ULA is used to estimate the precise DOAs of the interference signals, the interference and the noise powers, respectively [11]. The aforementioned beamforming algorithms significantly enhance the SINR performance of the coprime sensor array. Nevertheless, they require some complex calculations (such as spectral search and matrix inversion) and a large number of iterations, leading to an increased computational time and low computation efficiency in beamforming.

Fortunately, some deep learning methods are used to improve the computation efficiency and reduce the computation time of its powerful feature extraction and non-linear mapping ability [12]. A convolutional neural network (CNN)-based beamforming algorithm is presented to calculate the beamforming weight vector of the ULA. It takes less computation time than conventional algorithms for its weight sharing and feature extraction capability, which helps to speed up convergence in the training process [13]. On this basis, to further improve the robustness and the SINR performance in different interference environments, a two-stage beamforming algorithm is given [14]. Firstly, the CNN is used to estimate the interference signal. Then, the desired signal is acquired through a bidirectional long short-term memory (bi-LSTM) network, which contributes to improve the robustness and overall accuracy. Although the above methods can achieve excellent prediction accuracy and short computation times in the ULA, they are designed only for ULA and cannot make full use of the coprime feature, proved by coprime sensor arrays [14]. Therefore, the spatial resolution and interference suppression capability of the aforementioned deep learning algorithms remain to be improved.

In this paper, in order to maximize the utilization of the coprime sensor array feature and enhance the SINR performance, a robust adaptive beamforming algorithm based on an attention convolutional neural network (ACNN) is proposed, named the CAWE-ACNN algorithm. In this method, an ACNN model is established to enhance the relevant features contributing to beamforming weight vector estimation. Then, an INCM reconstruction algorithm is utilized to calculate the beamforming weight vector label. The INCM reconstruction algorithm utilizes a least squares method and a quadratic convex optimization problem to reconstruct the INCM and correct the mismatched SV, respectively. Subsequently, the sample covariance matrix received from the coprime sensor array and the calculated beamforming weight vector label is employed to train the presented ACNN module. Ultimately, the beamforming weight vector can be generated by the well-trained ACNN directly. The simulation results verify that the proposed algorithm achieves excellent SINR performance and high computation efficiency.

## 2. System Model

**Figure 1:**
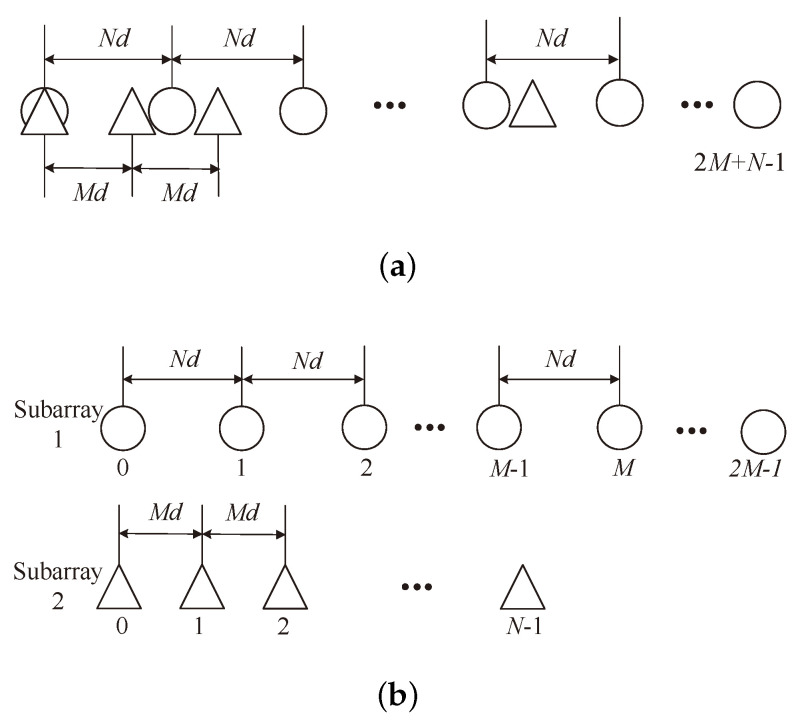
The coprime sensor array configuration. (**a**) The aligned coprime sensor array. (**b**) The two subarrays.

A coprime sensor array consisting of 2M+N−1 sensors is being considered, which is shown in Figure 1a. The coprime sensor array is composed of two ULAs, with one array containing 2M sensors and the other containing *N* sensors, where *M* and *N* are mutually prime integers with M<N. The two ULAs are named as subarray 1 and subarray 2. As shown in Figure 1b, subarray 1 and subarray 2 have inter-sensor spacings of Nd and Md. Here, the *d* is half of the wavelength.

Suppose that *K* narrowband signals are received by a coprime sensor array from ${\left\{\theta_{k}\right\}}_{k=1}^{K}$. The *l*-th snapshot of the received signal vector becomes $x(l)=a\left(\theta_{1}\right)s_{1}\left(l\right)+\sum_{k=2}^{K}a\left(\theta_{k}\right)s_{k}\left(l\right)+n\left(l\right)$, where $x(l)\in C^{(2M+N-1)\times 1}$, $\theta_{1}$ and $\theta_{k}$(k=2,…,K) represent the directions of the target signal and the K−1 interference signal, respectively. n(l) represents the Gaussian noise component. $s_{k}\left(l\right)$ is the *k*-th signal waveform. $a\left(\theta_{k}\right)={\left[1,e^{-j\frac{2\pi }{\lambda }x_{2}\sin\left(\theta_{k}\right)},\cdots ,e^{-j\frac{2\pi }{\lambda }x_{\left|H\right|}\sin\left(\theta_{k}\right)}\right]}^{T}$ [15] is the SV, where H={Mnd|0≤n≤N−1}∪{Nmd|0≤m≤2M−1}, containing $\left|H\right|=2M+N-1$ sensors. Here $x_{i}\in H$, $i=1,\ldots ,\left|H\right|$ represents the i-th position of the coprime sensor array with $x_{1}=0$. In addition, the beamformer output $y(l)=w^{H}x\left(l\right)$, where $w={\left[w_{1},\cdots ,w_{2M+N-1}\right]}^{T}$ represents the weight vector.

Let γ be the SINR of the coprime sensor array and defined as [16]

(1) $$\gamma =\frac{\sigma_{1}^{2}{\left|w^{H}a\left(\theta_{1}\right)\right|}^{2}}{w^{H}R_{i+n}w}$$

where $\sigma_{1}^{2}=E\left\{{\left|s_{1}\left(l\right)\right|}^{2}\right\}$ represents the desired signal power. $R_{i+n}=\sum_{k=2}^{K}\sigma_{k}^{2}a\left(\theta_{k}\right)a{\left(\theta_{k}\right)}^{H}+\sigma_{n}^{2}I_{2M+N-1}$ is the theoretical INCM, where $\sigma_{n}^{2}$ and ${\left\{\sigma_{k}^{2}\right\}}_{k=2}^{K}$ represent the powers of noise and interference signals, respectively.

Maximizing (1) can be seen as solving the following problem:

(2) $$\min_{w}w^{H}R_{i+n}w\;s.t.\;w^{H}a\left(\theta_{1}\right)=1$$

where $w^{H}a\left(\theta_{1}\right)=1$ ensures that the desired signal direction gain remains steady.

The approximation solution of problem (2) is given by [17]

(3) $$w_{mvdr}=\frac{{\hat{R}}^{-1}a\left(\theta_{1}\right)}{a^{H}\left(\theta_{1}\right){\hat{R}}^{-1}a\left(\theta_{1}\right)}$$

where $\hat{R}=\frac{1}{L}\sum_{l=1}^{L}x\left(l\right)x{\left(l\right)}^{H}$ represents sample covariance matrix, *L* represents the quantity of the snapshots.

## 3. Proposed CAWE-ACNN Algorithm

This section begins with detailed introduction to the ACNN framework. Subsequently, by a superior INCM reconstruction algorithm, we compute a near-optimal label. Ultimately, the well-trained ACNN is capable of accurately generating a near-optimal weight vector and achieving better SINR performance.

### 3.1. Structure of Proposed ACNN

**Figure 2:**
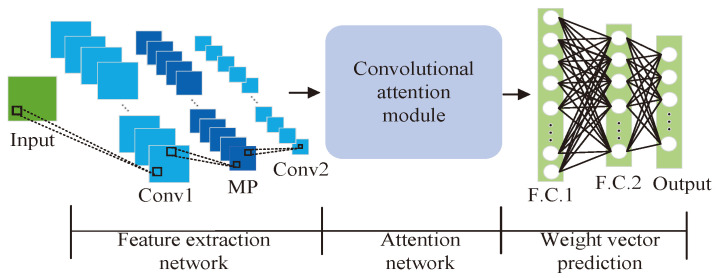
The proposed ACNN framework.

The problem of robust beamforming can be seen as a prediction problem in neural networks; that is, the neural network accepts the covariance matrix as input and maps it to the beamforming weight vector. Considering that the CNN is excellent in recognizing the spatial features of two-dimensional data, and the attention mechanism can be used to extract important features conducive to weight vector prediction, an ACNN framework is presented which is composed of a feature extraction network, an attention network and a weight vector prediction network. The structure of the ACNN is shown in Figure 2. The following content describes the hyperparameter setting for each network.

#### 3.1.1. Feature Extraction Network

This network is employed to learn crucial features that contribute to reduce data dimensions and estimate weight vectors. The first layer is an input layer of size (2M+N−1)×(2M+N−1)×3 (the generation of the input data will be given in Section 3.3). The convolutional layers are structured as second and fourth layers, each with 32 and 64 feature maps of dimensions 3×3, which employ exponential linear units (ELUs) as activation functions. The third layer, serving as the max-pooling layer, with feature maps sized 2×2, plays a role in parameter reduction within the network.

#### 3.1.2. Attention Network

**Figure 3:**
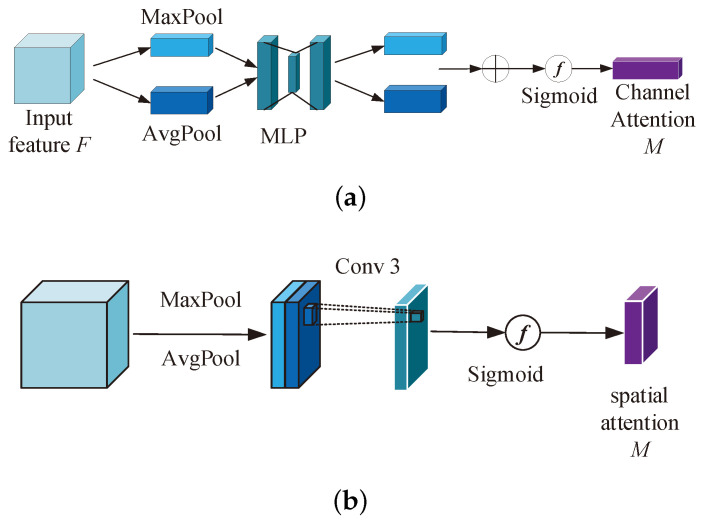
The convolutional attention unit. (**a**) The channel attention module. (**b**) The spatial attention module.

To enhance the performance of coprime sensor array weight vector estimation, an attention network is introduced which is called convolutional attention module. The convolutional attention module has two parts: channel and spatial attention units, which are illustrated in Figure 3.

The convolutional layer comprises multiple output channels. Each channel exerts varying degrees of influence on the performance of beamforming weight vector estimation. So, it is essential to employ the channel attention unit to assess various channels and assign the vital channels with larger weights. As can be seen from Figure 3a, the channel attention unit can be mathematically represented by

(4) $$\begin{array}{cc} M_{c}\left(F\right)= & \sigma (MLP(AvgPool(F))+MLP(MaxPool(F)))\\ = & \sigma (W_{1}\left(W_{0}\left(F_{avg}^{c}\right)\right)+W_{1}\left(W_{0}\left(F_{max}^{c}\right)\right)) \end{array}$$

where σ represents the sigmoid function. MLP(·) stands for multi-layer perception with two hidden layers, which constitutes a shared network architecture. $F\in R^{D\times H\times W}$ denotes an input feature, where H×W and *D* are the spatial dimension and channel dimension, respectively. MaxPool(·) and AvgPool(·) are the maximum and average pooling operations designed to integrate the spatial information of F. $F_{max}^{c}$ and $F_{avg}^{c}$ denote the maximum and average pooled features, respectively. In addition, $W_{0}\in R^{D/r\times D}$ and $W_{1}\in R^{D\times D/r}$ are the weights of the two hidden layers in the multi-layer perception. And *r* represents the reduction ratio.

The spatial attention module concentrates on the important spatial information of the input features. As we can see from Figure 3b, the spatial attention unit can be mathematically represented by

(5) $$\begin{array}{cc} M_{s}\left(F\right)= & \sigma (f^{7\times 7}\left(\left[AvgPool(F);MaxPool(F)\right]\right))\\ = & \sigma (f^{7\times 7}\left(\left[F_{avg}^{s};F_{max}^{s}\right]\right)) \end{array}$$

where $f^{7\times 7}$ represents a convolutional operation with a feature map of size 7×7. $F_{avg}^{s}$ and $F_{max}^{s}$ denote average and max pool features, respectively, and are derived through the aggregation of channel information.

To sum up, the attention mechanism can be used to extract important channel and spatial features conducive to weight vector prediction. Here, the channel feature represents the data correlation between different channels. By extracting the data correlation between different channels, the importance of different channels is evaluated via the channel attention unit, and the vital channels are assigned with larger weights. The spatial feature is the amplitude and phase information of the input signal. By extracting amplitude and phase information, the space attention unit helps to enhance the desired signal and suppress the interference signal. Therefore, the SINR performance of the proposed algorithm is improved.

#### 3.1.3. Weight Vector Estimation Network

The weight vector prediction module is composed of one output layer and two fully connected layers. Its purpose is to map the weight information to the weight vector of the coprime sensor array. The output layer, comprising 2(2M+N−1) neurons, is responsible for estimating the weight vector (in Section 3.3, we will introduce the preprocessing of the output data). Before the output layer, two fully connected (FC) layers are employed, which comprise 128 and 32 neurons, respectively. The output layer utilizes a linear activation function to estimate the beamforming weight vector.

### 3.2. Weight Vector Label Generation

This section delves into the process of generating the label. Initially, we acquire a CV-ULA by inferring virtual sensors, and then employ the spatial smoothed matrix of the CV-ULA to estimate the DOA. Following this, a least squares problem is employed to estimate the power of the interference signal; subsequently, the INCM is computed. Afterwards, the target signal is rectified via a quadratic convex optimization problem. At last, the beamforming weight vector label for the coprime sensor array is utilized to train the ACNN model.

#### 3.2.1. DOA Estimation for Label Generation

As we all know, the virtual array can expand the array aperture. A CV-ULA is calculated by extracting the continuous locations of the virtual element from −MNd to MNd. To ensure the precise estimation of the DOA, we construct a spatially smoothed matrix $R_{ss}=\frac{1}{g}\sum_{p=1}^{g-1}J_{p}z_{v}z_{v}^{H}J_{p}^{H}$ [11], where $J_{p}=\left[0_{g\times (g-1-p)}\;I_{g\times g}\;0_{g\times p}\right]\in {\left\{0,1\right\}}^{g\times (2g-1)}$ represents a selection matrix, g=(2MN+1)/2. In particular, $z_{v}$ denotes the observation vector of the CV-ULA.

By the spatial smoothed matrix $R_{ss}$, the multiple signal classification (MUSIC) spatial spectrum is as follows [18]:

(6) $$P\left(\theta \right)=\frac{1}{d{\left(\theta \right)}^{H}VV^{H}d\left(\theta \right)}$$

where d(θ) represents the SV of the CV-ULA when the sensors are distributed from 0 to MNd. $\theta \in [-90^{\circ },90^{\circ }]$ represents the hypothetical direction and V denotes the noise subspace of $R_{ss}$.

Utilizing (6), we can identify the directions ${\left\{{\hat{\theta }}_{k}\right\}}_{k=1}^{K}$ of all signals by searching the spectrum peaks. Specifically, according to the given spectral function, the estimate of the DOA can be obtained by seeking peak values. Utilizing the estimated DOAs, the reconstruction of the INCM and the estimation of the target signal SV are finished.

#### 3.2.2. INCM Reconstruction

Apart from estimating the DOA of interference, it is essential to account for the powers of interference and noise when reconstructing the INCM. Hence, a least squares problem is shown as follows:

(7) $$\min_{\Lambda }{∥\hat{R}-\sigma_{n}^{2}I-A\left(\hat{\theta }\right)\Lambda A^{H}\left(\hat{\theta }\right)∥}_{F}^{2}\;s.t.\;\Lambda >0$$

where $\Lambda =\mathrm{diag}(\left[\sigma_{1}^{2},\sigma_{2}^{2},\cdots ,\sigma_{K}^{2}\right])$ stands for the signal powers. $A\left(\hat{\theta }\right)=[a\left({\hat{\theta }}_{1}\right),a\left({\hat{\theta }}_{2}\right),$$\cdots ,a\left({\hat{\theta }}_{K}\right)]\in C^{(2M+N-1)\times K}$ is the SV matrix. The noise power $\sigma_{n}^{2}$ can be approximately computed by $\lambda_{min}\left(\hat{R}\right)$, which stands for the minimum eigenvalue of $\hat{R}$.

Hence, the solution to (7) is ${\left(B^{H}B\right)}^{-1}B^{H}c$, in which $B=\{\mathrm{vec}\left(a\left({\hat{\theta }}_{1}\right)a^{H}\left({\hat{\theta }}_{1}\right)\right),\cdots ,$$\mathrm{vec}(a\left({\hat{\theta }}_{K}\right)a^{H}\left({\hat{\theta }}_{K}\right))\}$ and $c=\mathrm{vec}(\hat{R}-\sigma_{n}^{2}I_{2M+N-1})$. So, the INCM undergoes a modification as follows:

(8) $${\hat{R}}_{i+n}=\sum_{k=2}^{K}{\hat{\sigma }}_{k}^{2}a\left({\hat{\theta }}_{k}\right)a^{H}\left({\hat{\theta }}_{k}\right)+\sigma_{n}^{2}I_{2M+N-1}$$

where ${\hat{\sigma }}_{k}^{2}$ represents the estimated interference signal powers.

#### 3.2.3. Estimation of Target Signal SV

From (3), the exact target signal SV is necessary for calculating the beamforming weight vector. Therefore, the SV of the desired signal is corrected through the optimization problem below [19]

(9) $$\begin{array}{cc} \min_{e} & \;{\left(\bar{a}\left({\hat{\theta }}_{1}\right)+e_{⊥}\right)}^{H}{\hat{R}}^{-1}\left(\bar{a}\left({\hat{\theta }}_{1}\right)+e_{⊥}\right)\\ s.t.\; & {\bar{a}}^{H}\left({\hat{\theta }}_{1}\right)e_{⊥}=0\\ & {\left(\bar{a}\left({\hat{\theta }}_{1}\right)+e_{⊥}\right)}^{H}UU^{H}\left(\bar{a}\left({\hat{\theta }}_{1}\right)+e_{⊥}\right)\leq {\bar{a}}^{H}\left({\hat{\theta }}_{1}\right)UU^{H}\bar{a}\left({\hat{\theta }}_{1}\right) \end{array}$$

where $e_{⊥}$ is the orthogonal component of the error steering vector e between the exact desired SV $a\left({\hat{\theta }}_{1}\right)=\bar{a}\left({\hat{\theta }}_{1}\right)+e_{⊥}$ and the estimated desired SV $\bar{a}\left({\hat{\theta }}_{1}\right)$. The columns of U consist of K−1 eigenvectors corresponding to the smallest eigenvalues of the matrix $C=\int_{\Theta }a\left(\theta \right)a^{H}\left(\theta \right)\;d\theta$. The orthogonality between $e_{⊥}$ and $\bar{a}\left({\hat{\theta }}_{1}\right)$ is guaranteed by the equality constraint ${\bar{a}}^{H}\left({\hat{\theta }}_{1}\right)e_{⊥}$ = 0.

The desired signal SV can be restated as $\hat{a}\left({\hat{\theta }}_{1}\right)=\bar{a}\left({\hat{\theta }}_{1}\right)+{\hat{e}}_{⊥}$ by solving the optimization problem (9), where ${\hat{e}}_{⊥}$ represents the estimated orthogonal component.

Substituting $\hat{a}\left({\hat{\theta }}_{1}\right)$ and ${\hat{R}}_{i+n}$ into (3), the weight vector is shown as follows:

(10) $$w^{\mathrm{label}}=\frac{{\hat{R}}_{i+n}^{-1}\hat{a}\left({\hat{\theta }}_{1}\right)}{{\hat{a}}^{H}\left({\hat{\theta }}_{1}\right){\hat{R}}_{i+n}^{-1}\hat{a}\left({\hat{\theta }}_{1}\right)}$$

where ${\hat{R}}_{i+n}$ is the exact INCM.

### 3.3. Training and Testing Process of ACNN

Consider X as a real-valued input datum. The first input channel consists of the absolute values of the sample covariance matrix elements $\hat{R}$ as ${\left[{\left[X\right]}_{:,:,1}\right]}_{i,j}=\left|{\left[\hat{R}\right]}_{i,j}\right|$. The second input channel comprises the imaginary components of the sample covariance matrix $\hat{R}$, while the third input channel consists of the real components, the form is as follows: ${\left[{\left[X\right]}_{:,:,3}\right]}_{i,j}=\mathrm{Imag}\left\{{\left[\hat{R}\right]}_{i,j}\right\}$ and ${\left[{\left[X\right]}_{:,:,2}\right]}_{i,j}=\mathrm{Real}\left\{{\left[\hat{R}\right]}_{i,j}\right\}$. In order to speed up convergence, the X will be normalized.

By decomposing the elements into the real and imaginary components, the beamforming weight vector $w^{\mathrm{label}}=[\mathrm{Real}\left\{w_{1}^{\mathrm{label}}\right\},\mathrm{Imag}\left\{w_{1}^{\mathrm{label}}\right\},\cdots ,\mathrm{Real}\left\{w_{\left|H\right|}^{\mathrm{label}}\right\},$ $\mathrm{Imag}\{w_{\left|H\right|}^{\mathrm{label}}\}]$, where $\left|H\right|=2M+N-1$ and $w^{\mathrm{label}}\in C^{2\left|H\right|\times 1}$.

The ACNN is supplied with the covariance matrix in the training phase to acquire the capability of estimating the weight vector, with the mean squared error serving as the loss function. Subsequently, the weight vectors are predicted by the well-trained ACNN in the testing phase.

### 3.4. Summary of Proposed Algorithm

Here is a summary of the proposed CAWE-ACNN algorithm.

(1) Establish the ACNN model.
(2) Collect received signal samples $X=[x_{1},\cdots ,x_{q},\cdots ,x_{Q}]$ with signal sources positioned at various DOAs and SNRs, where *Q* represents the quantity of signal samples x in the data set.
(3) Acquire the sample covariance matrix samples $\bar{R}=\left[{\hat{R}}_{1},\cdots ,{\hat{R}}_{q},\cdots ,{\hat{R}}_{Q}\right]$ and compute the beamforming weight vector samples $W^{\mathrm{label}}=\left[w_{1}^{\mathrm{label}},\cdots ,w_{q}^{\mathrm{label}},\cdots ,w_{Q}^{\mathrm{label}}\right]$ by (10).
(4) Preprocess the sample data to obtain the training data.
(5) Train the ACNN using the training data.
(6) The beamforming weight vector $w^{\mathrm{predict}}$ is predicted by the well-trained ACNN.

## 4. Simulation Results

Simulations are conducted to prove the effectiveness of the proposed beamforming algorithm for coprime sensor arrays with M=3 and N=5. All the experiments are performed on the same computer (Intel(R) Core(TM) i7-6700 CPU @ 3.40 GHz produced by DELL). The proposed CNN framework is realized and trained by PyCharm 2020.2.3, based on TensorFlow 1.14.0 and Keras 2.3.1, on this computer with a CPU. During the training stage, one desired and two interference signals are ultilized to train the proposed ACNN. The DOA of the target signal varies from $\left[-30^{\circ },30^{\circ }\right]$ with step of $1^{\circ }$. The DOAs of two interference signals vary from $\left(-90^{\circ },-30^{\circ }\right)$ and $\left(30^{\circ },90^{\circ }\right)$ with step of $2^{\circ }$, respectively. In the testing stage, the desired signal is positioned at $\theta_{0}=5^{\circ }$ and the two interferences are positioned at $\theta_{1}=-20^{\circ }$ and $\theta_{2}=40^{\circ }$ with 30 dB interference-to-noise-ratios (INR), respectively. The number of snapshots K=20. In total, 500 Monte Carlo experiments are utilized to calculate the output SINR. The detail parameter setting of the ACNN module and the training process are presented in Table 1. The proposed beamformer is compared to the following beamformers: the diagonal loading sample matrix inversion (DLSMI) method, the sequential quadratic programming (SQP) algorithm, the CNN algorithm and the CA-CMR method. For DLSMI beamformer, the loading factor is set to be tenth the noise power. δ=0.1 and eight principal eigenvectors of matrix C for the SQP beamformer.

### 4.1. Mismatch Due to DOA Estimation Error

A scenario of random errors in DOA estimation is taken into account. These errors follow a uniform distribution within $\left[-2^{\circ },2^{\circ }\right]$.

**Figure 4:**
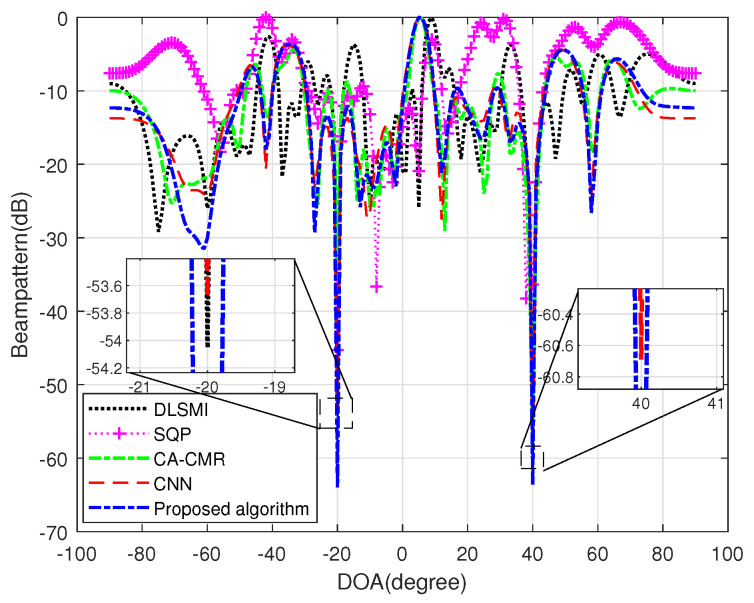
Beampattern of different algorithms under condition of DOA estimation error.

Figure 4 illustrates the beampatterns generated by various algorithms under the condition of DOA estimation error. It is evident that even a minor error in DOA estimation can result in a significant increase in sidelobe level for DLSMI and SQP. The other algorithms can place nulls at the interference direction and their main lobes are close to the true desired signal direction. Specifically, the proposed algorithm not only positions the deepest nulls at the interference directions, but also preserves an undistorted response at the desired signal direction in the case of DOA estimation error. This validates that the proposed method is robust to DOA estimation error.

**Figure 5:**
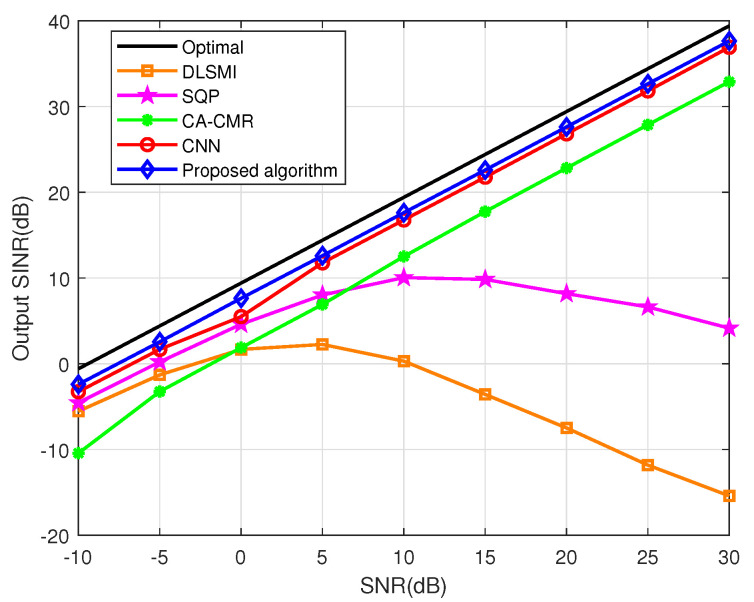
SINR vs. SNR under condition of DOA estimation error.

The SINR versus SNR of different algorithms within DOA estimation error is evaluated in Figure 5. In Figure 5, when the SNR changes from −10 dB to 30 dB, except for DLSMI and SQP algorithms, the output SINRs of other algorithms increase gradually. Specially, the performance of the proposed methods surpasses other comparative algorithms in terms of the output SINR. This is because the proposed ACNN can fully extract channel and spatial features by introducing the spatial and channel attention units. And the simulation experiment indicates that the proposed ACNN is helpful for accurately estimating the beamforming weight vector.

**Figure 6:**
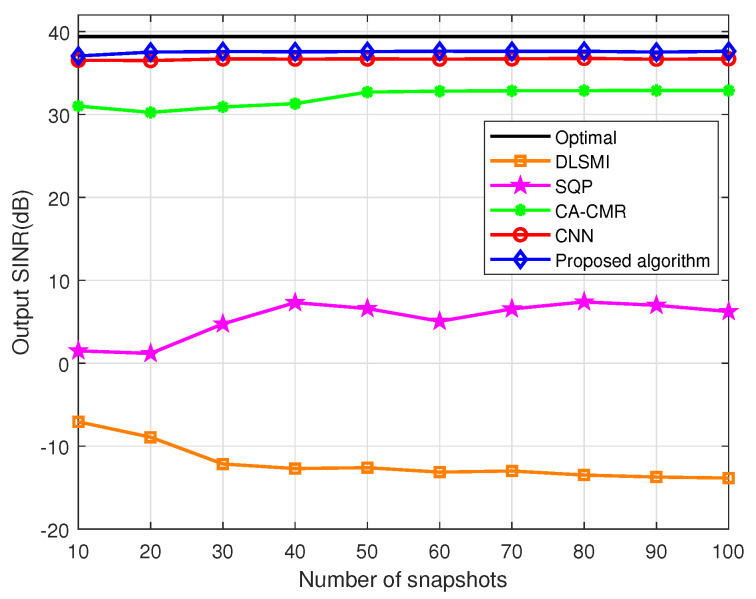
SINR vs. quantity of snapshots within DOA estimation error.

The SINR versus the quantity of the snapshots within the DOA estimation error is evaluated in Figure 6. The SNR of the desired signal remains constant at 30 dB. The SINRs of the DLSMI and the SQP algorithm fluctuate when the snapshots vary from 10 to 100. It is evident that the proposed algorithm outperforms other algorithms in terms of SINR performance. This is primarily attributed to the exceptional weight vector label and the outstanding weight vector estimation capability of the proposed ACNN network.

### 4.2. Mismatch Due to Sensor Position Error

Assume that the sensor positional error variable is uniformly distributed in [−0.025λ, 0.025λ], where λ represents wavelength.

**Figure 7:**
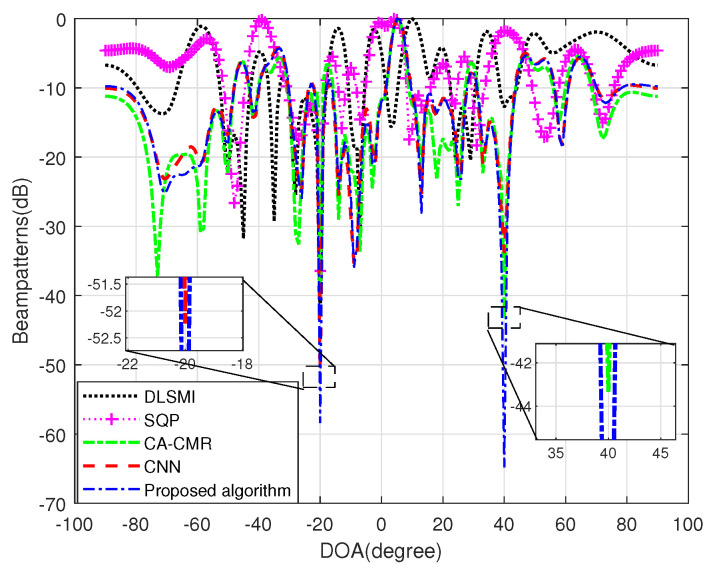
Beampattern of different algorithms within sensor position error.

Figure 7 illustrates the beampatterns generated by various algorithms within sensor position errors. It can be seen from Figure 7 that the CNN algorithm, CA-CMR algorithm and the proposed algorithms can all keep the main lobe of the beampattern in the desired signal direction, protecting the power of the expected signal from being consumed, and at the same time can place nulls at the interference directions. Among all the algorithms, the proposed CAWE-ACNN algorithm has a better directional pattern performance. Specifically, it can not only guarantee the reception of the desired signal, but also has the deepest nulls in two interference directions. It validates that the proposed CAWE-ACNN algorithm is robust when the SV of the desired signal is mismatched due to the sensor position error.

**Figure 8:**
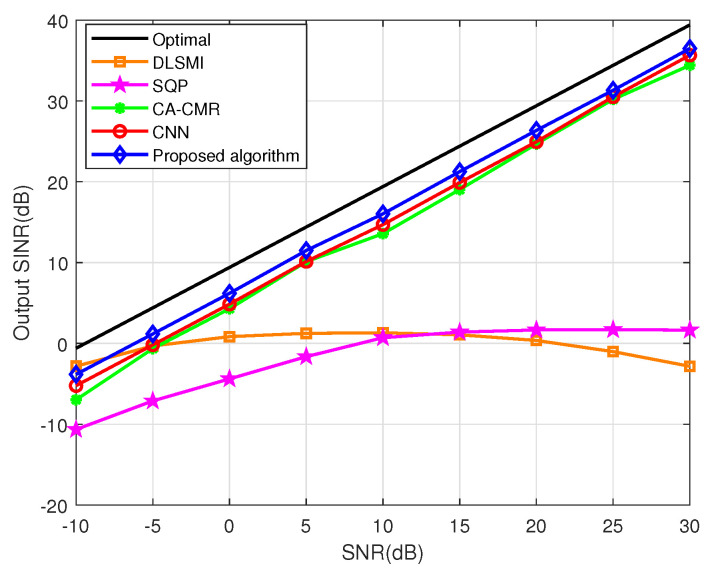
SINR vs. SNR in the case of sensor position error.

The SINR versus SNR of different algorithms within sensor position error is shown in Figure 8. From Figure 8, as the SNR increases from −10dB to 30dB, the output SINRs of the CNN, CA-CMR and proposed algorithms increase steadily. The SQP algorithm tends to become steady, and the DLSMI algorithm increases firstly and then decreases. Specifically, the proposed algorithm shows superior SINR performance compared to other algorithms under the majority of the range of SNRs. The reason is that the proposed ACNN can fully extract spatial and channel features of the covariance matrix.

**Figure 9:**
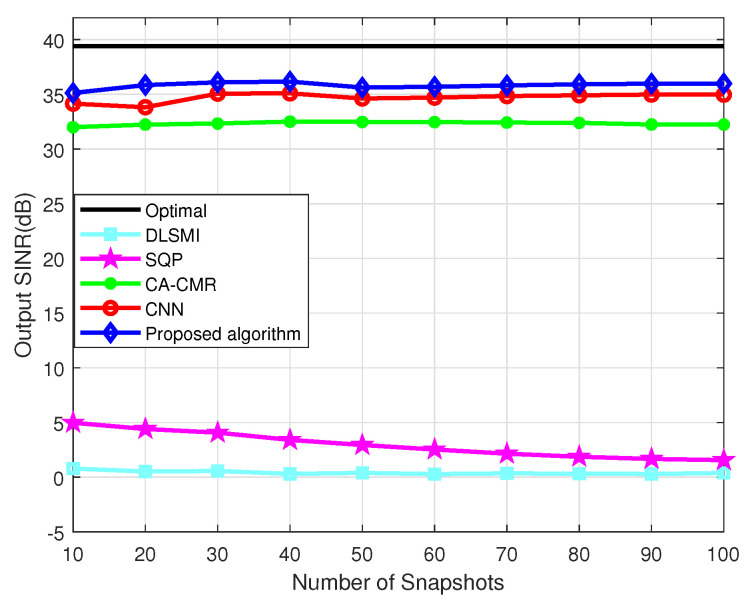
SINR vs. number of snapshots within sensor position error.

Figure 9 illustrates the SINR versus the number of snapshots of different beamformers when the sensor position error exists. From Figure 9, the output SINR curves of all the comparison algorithms are steady. Specifically, the SINR of the proposed CAWE-ACNN algorithm is higher than the other comparison algorithms in a different number of snapshots. This is mainly because the proposed algorithm has an excellent beamforming weight vector label, which helps to improve the prediction performance of ACNN. Although the SQP algorithm has corrected the error of the desired signal steering vector, its output performance is only better than the DLSMI algorithm, yet lower than the CA-CMR algorithm.

### 4.3. Computation Complexity Analysis

The computational complexity of the proposed algorithm mainly includes the following: (1) the problem (9) for SV estimation, of the order $O({\left(M+N\right)}^{3.5})$, and the reconstruction of the INCM, of the order $O({\left(M+N\right)}^{2}Z)$, where *Z* denotes the number of samples. (2) The computational complexity of the ACNN module: According to the ACNN model, the computational complexity is mainly generated by the convolution layers (in the feature extracting network and the space attention unit) and the full connection layers (in the weight vector prediction network and the channel attention unit). Therefore, the computational complexity of the ACNN model is $O(P{\left(M+N\right)}^{2})$, where *P* is related to the parameter of the ACNN.

Table 2 compares the complexity of the proposed CAWE-ACNN, the DLSMI algorithm, the SQP algorithm, the CNN algorithm and the CA-CMR algorithm, where *S* and *L* represent the number of sampling points in Θ and hypothetical directions. The results indicate that the computation complexity of the CAWE-ACNN algorithm is almost equivalent to the CNN algorithm, but lower than that of the CA-CMR method.

### 4.4. Computation Time Analysis

The computation time of the beamforming algorithms is shown in Table 3. Here, the computation time is calculated from feeding 120 sample covariance matrixes to generate the weight vectors. It is evident that the proposed method exhibits less computation time than the SQP and CA-CMR methods. This is because the proposed algorithm avoids both matrix inversion and spectrum search process. Although the DLSMI algorithm and the CNN algorithm take less computation time than the proposed algorithm, the SINRs performances of the two methods are limited compared to the proposed method.

### 4.5. FLOPs Analysis

Table 4 represents the FLOPs of different algorithms. It can be seen that the SQP and CA-CAM algorithms require the largest number of FLOPs, while the FLOPs of the proposed algorithm are higher than that of the DLSMI and CNN algorithms. But the proposed method achieves a better SINR performance than the DLSMI and CNN algorithms. This is because the attention module is used in the proposed ACNN to improve the fitting ability and the SINR performance, which is inevitable when increasing computation complexity.

### 4.6. Computation Efficiency

Based on the FLOPs analysis and the computation time of the algorithms, a formula for computational efficiency is defined as [20]

(11) CE=F/T

where *F* denotes the number of FLOPs and *T* stands for the one-computation time of the algorithm. CE represents the number of FLOPs performed by the beamforming algorithm per unit time, which reflects the computation performance of the algorithm.

**Figure 10:**
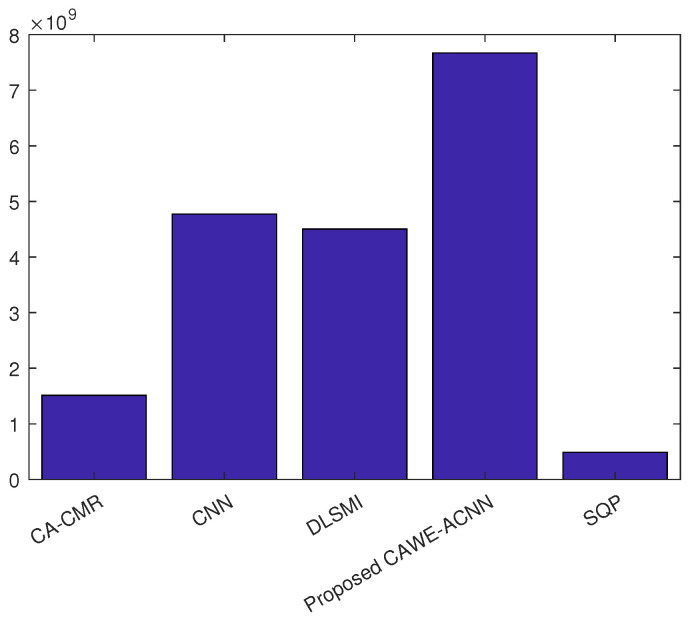
Computation efficiency of different algorithms.

Based on the formulation (11), the computation efficiency of the DLSMI algorithm, the SQP algorithm, the CNN algorithm, the CA-CMR algorithm and the proposed CAWE-ACNN algorithm are calculated in Figure 10. From Figure 10, it can be seen that the proposed CAWE-ACNN algorithm achieves the best computation efficiency than other compared beamformers. This is because the proposed CAWE-ACNN algorithm possesses strong computational and representational abilities, which enhance its efficiency in extracting features and processing complex data, thereby greatly simplifying the process of beamforming.

## 5. Conclusions

In this paper, a CAWE-ACNN algorithm is designed for adaptive beamforming in coprime sensor arrays. Initially, an ACNN module composed of a spatial attention unit and a channel attention unit is established to improve the SINR performance. Then, the beamforming weight vector label is obtained through an INCM reconstruction algorithm. Based on the calculated beamforming weight vector, the training process of the proposed ACNN is completed. Ultimately, the well-trained ACNN is capable of accurately and efficiently outputting the beamforming weight vector. The simulation results validate the superior SINR performance and high computation efficiency of the proposed beamformer method compared to other beamforming methods. However, there are still some issues that require further study and improvement. The coprime sensor array used in this paper is still a linear array with limited coverage and angular resolution capabilities. In the future, this algorithm can be extended to a planar array to receive or transmit signals with a wider spatial range and have better angular resolution.

## Figures and Tables

**Table 1:** Hyperparameter setting of the proposed algorithm.

Parameter		Value
input		(2M+N−1)×(2M+N−1)×3
Conv1		32×3×3
MP		2×2
Conv2		64×3×3
Conv3		1×7×7
MLP	$W_{0}$	(C/r)×C
	$W_{1}$	C×(C/r)
FC1		128
FC2		32
Output		2(2M+N−1)
Epochs		500
Learning rate		0.001
Batch size		32
Loss		MSE
Optimizer		Adam

**Table 2:** Computation complexity of different beamformers.

Beamforming Algorithms	Computation Complexity
Proposed CAWE-ACNN	$O(P{\left(M+N\right)}^{2})$
DLSMI [3]	$O({\left(M+N\right)}^{3})$
SQP [6]	$O({\left(M+N\right)}^{3.5}S)$
CNN [13]	$O(K{\left(M+N\right)}^{2})$
CA-CMR [11]	$O(L{\left(M+N\right)}^{3.5})$

**Table 3:** Computation time of different beamformers.

Beamforming Algorithms	Computation Time
Proposed CAWE-ACNN	0.1372 s
DLSMI [3]	0.0381 s
SQP [6]	7.5748 s
CNN [13]	0.1084 s
CA-CMR [11]	5.6087 s

**Table 4:** FLOPs of different algorithms.

Beamforming Algorithms	FLOPs
Proposed CAWE-ACNN	8.77 M
DLSMI [3]	1.43 M
SQP [6]	30.87 M
CNN [13]	4.31 M
CA-CMR [11]	70.67 M

## Data Availability

The data presented in this study are available on request from the corresponding author.
